# Supplementary material for: IntLIM: integration using linear models of metabolomics and gene expression data
Source: BMC Bioinformatics. 2018 Mar 5;19:81. doi: 10.1186/s12859-018-2085-6 (PMC5838881; doi:10.1186/s12859-018-2085-6)
Supplement: Supplementary file 1 — Figure S3. “Volcano plots” of Spearman correlation differences vs. FDR- adjusted p-values (of interaction term in linear model, see Methods) for A) NCI-60 cell line analysis and B) breast cancer data analysis. (PDF 604 kb) [file 12859_2018_2085_MOESM1_ESM.pdf]

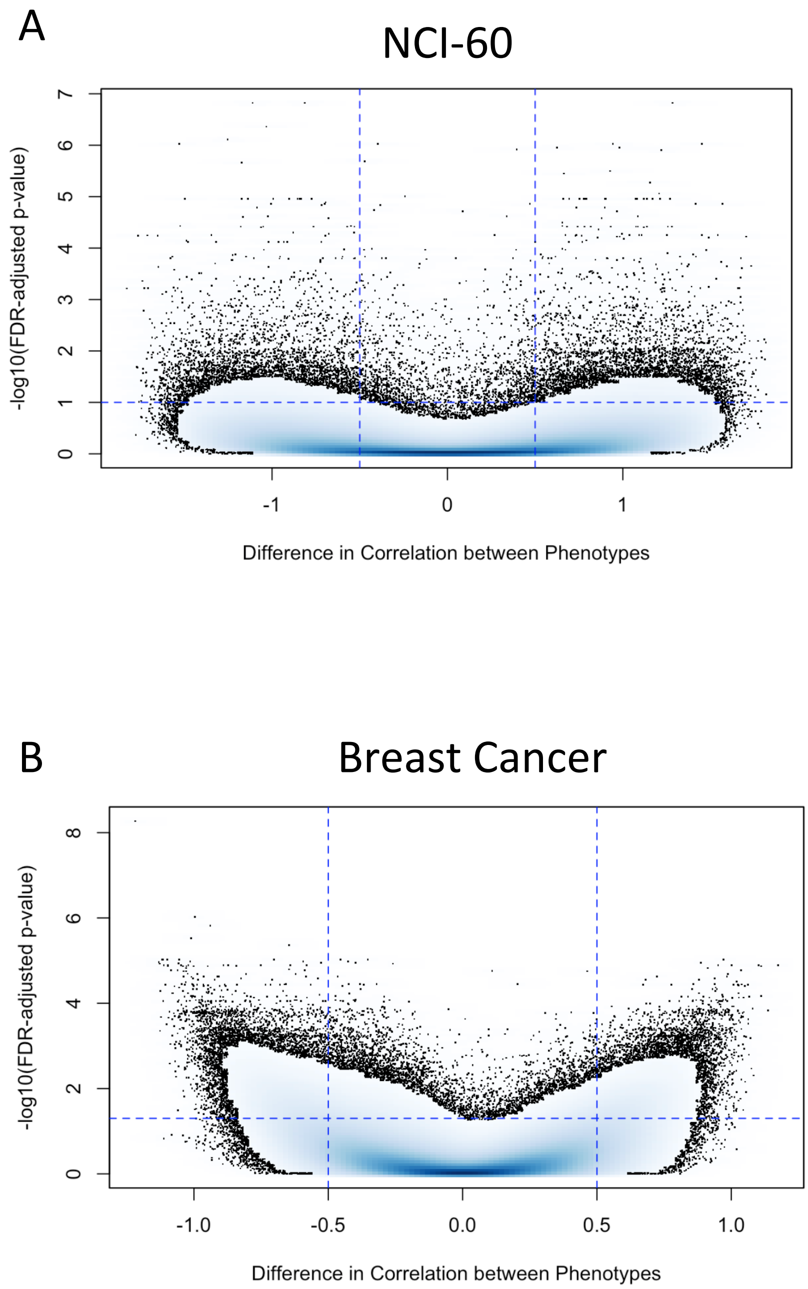

Figure S3: **"**Volcano plots" of Spearman correlation differences vs. FDR- adjusted p-values (of interaction term in linear model, see Methods) for A) NCI-60 cell line analysis and B) breast cancer data analysis.
