# Supplementary material for: IntLIM: integration using linear models of metabolomics and gene expression data
Source: BMC Bioinformatics. 2018 Mar 5;19:81. doi: 10.1186/s12859-018-2085-6 (PMC5838881; doi:10.1186/s12859-018-2085-6)
Supplement: Supplementary file 2 — Documentation on IntLIM. (DOCX 1444 kb) [file 12859_2018_2085_MOESM2_ESM.docx]

**IntLIM Documentation**

# Summary

Interpretation of metabolomics data is very challenging. Yet it can be eased through integration of metabolmoics with other ‘omics’ data. The IntLIM package, which includes a user-friendly RShiny web interface, aims to integrate metabolomic data with transcriptomic data. We implement a simple linear modeling approach to integration, where we focus on understanding how specific gene-metabolite associations are affected by phenotypic features. To this end, we include an interaction term in our linear model that specifically tests whether a gene-metabolite association differs by phenotype. The overall workflow involves the following steps:

1) input gene expression/metabolomics data files,

2) filter data sets by gene and metabolite abundances and imputed values,

3) run linear models on all possible gene-metabolites pairs and extract FDR-adjusted interaction p-values,

4) filter results by FDR-adjusted p-values and Spearman correlation differences,

5) plot/visualize user-defined gene-metabolite associations

The package, source code, vignettes, and formatted datasets used in to produce results in this manuscript are available for download on GitHub (https://github.com/mathelab/IntLIM).

# Installing and Running IntLIM

**Installing IntLIM**

The “devtools” package [1] is the simplest way to directly install IntLIM:

install.packages(devtools)

library(devtools)

install_github(“mathelab/IntLIM”)

In some cases, it may be necessary to install the Bioconductor package *MultiDataSet* (Hernandez-Ferrer et al, 2017):

## try http:// if https:// URLs are not supported

source("https://bioconductor.org/biocLite.R")

biocLite("MultiDataSet")

**Running IntLIM**

The IntLIM package provides the necessary functions to carry out the workflow of integrating gene expression and metabolomics data and finding phenotype-dependent gene-metabolite associations. The information below is meant to provide an overview of the workflow and the functions available in the package. Details on usage and parameters available for each function are included in the package documentation provided by each function. This documentation is accessed by typing “?functionname”, where “functionname” is the name of the function, such as ReadData, FilterData, etc.

**Step1: Read in Data**

Through the ReadData() function, users input a comma-separated-values (CSV) file that contains information on the input files. This input CSV file must contain 2 columns and 6 rows and must include the following:

type,filenames

metabData,myfilename

geneData,myfilename

metabMetaData,myfilename (optional)

geneMetaData,myfilename (optional)

sampleMetaData,myfilename

The “myfilename” represent file names for the respective data types without the file directory, which is assumed to be the same as the input file. **Thus, IntLIM assumes that all input files are in the same directory.** The file names for the gene data (‘geneData’) and metabolite data (‘metabData’) must be normalized and can be optionally transformed (if not transformed, the ReadData() has a parameter to apply log2 transformation). Sample meta-data (‘sampleMetaData’) must include at least one phenotype column (to calculate the linear model interaction term). Meta-data for genes and metabolites (e.g. names, alternate ids, associate pathways) are optional. The gene expression data, gene meta-data, and sample meta-data are input into an ExpressionSet object. The metabolite abundance data, metabolite meta-data, and sample meta-data are input into a new MetaboliteSet object, a new eSet object designed to contain metabolomics data [2]. Both objects are integrated into a MultiDataSet object- a multi-‘omics object allowing integration of eSet objects from different ‘omics data sets.

**Step2: Filter/Observe Data**

The FilterData() function filters data by percentile of gene expression/metabolite abundances as well as by percent of missing or imputed metabolite values.

The ShowStats() function summarizes the metabolite and gene expression data by number of genes, metabolites, and samples for each set as well as common samples. The PlotDistribution() function allows users to produce a boxplot of the distribution of gene expression and metabolite abundance data (Figure 1).

**
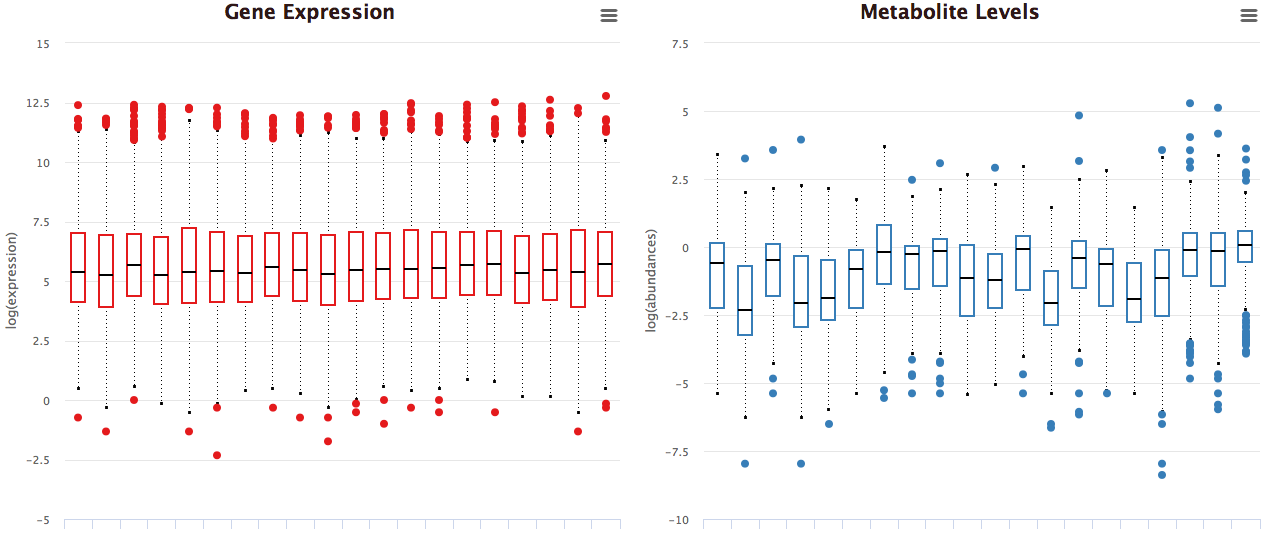
**

Figure 1: Example distribution of genes and metabolites

Prior to running the model, the user can also perform a principal component analysis of the gene expression and metabolite data using the PlotPCA() function (Figure 2). The ‘stype’ command allows the user to select a column from the sample meta data that color-codes the samples into two categories (two cancer types, tumor vs. non-tumor, etc).

**
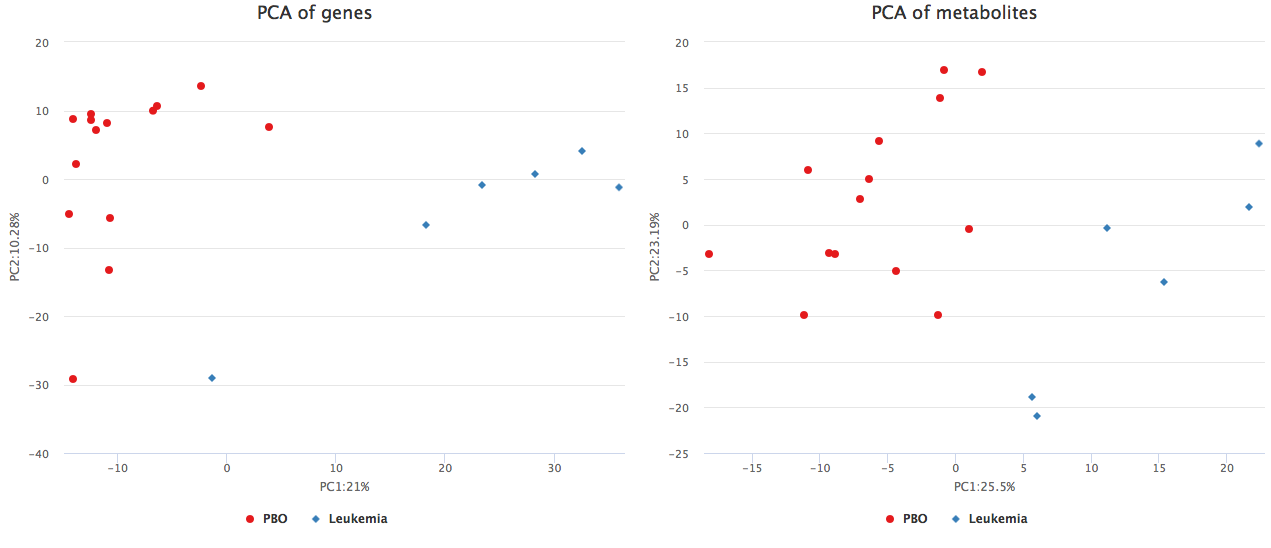
**

Figure 2: Example principal component analysis plots

**Step3: Run IntLIM**

The linear models are run by the RunIntLim() function. The ‘stype’ command allows the user to select a column from the sample meta data for the two categories to be compared (two cancer types, tumor vs. non-tumor, etc). Currently, IntLIM only supports comparison of two categories. The resulting object from the analysis is an IntLimResults object containing slots for un-adjusted and False Discovery Rate (FDR)-adjusted p-values for the interaction coefficient. A significant FDR-adjusted p-value implies that the slope of gene-metabolite association in one phenotype is different from the other. The RunIntLim function is based heavily on the MultiLinearModel functions developed for the ClassComparison package part of oompa (http://oompa.r-forge.r-project.org). The DistPValues() function allows the user to observe a histogram of the p-values prior to adjustment (Figure 3). The pvalCorrVolcano() function allows users to observe a volcano plot comparing the Spearman correlation difference between groups to the –log10(FDR-adjusted p-value) (Figure 4).

**
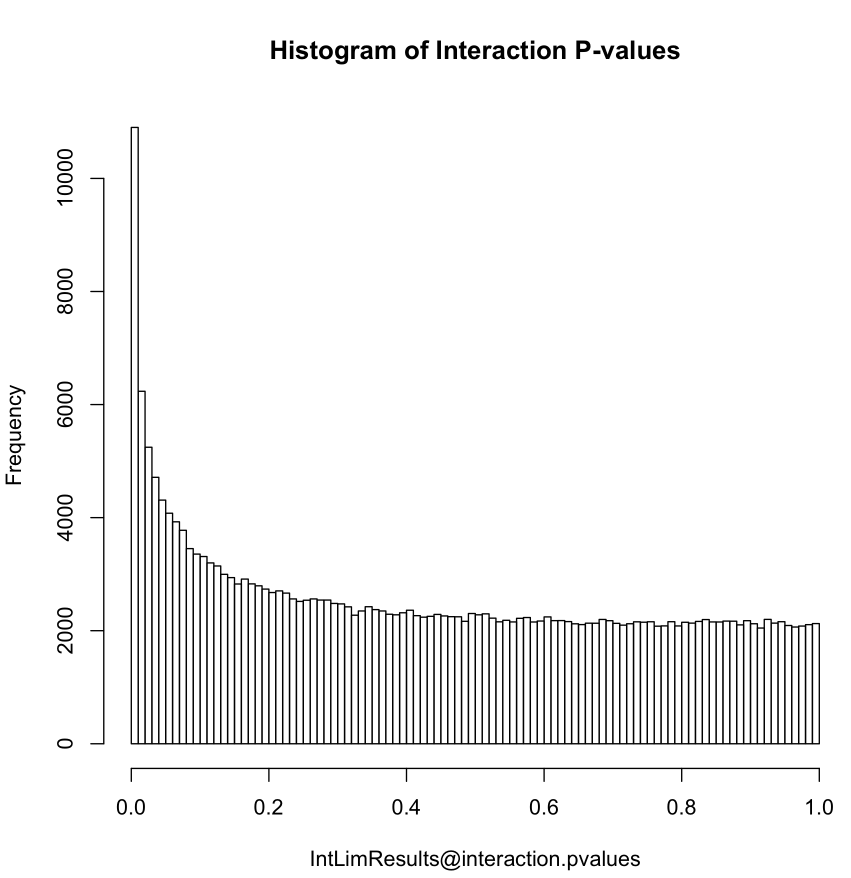
**

Figure 3: Example p-values histogram

**
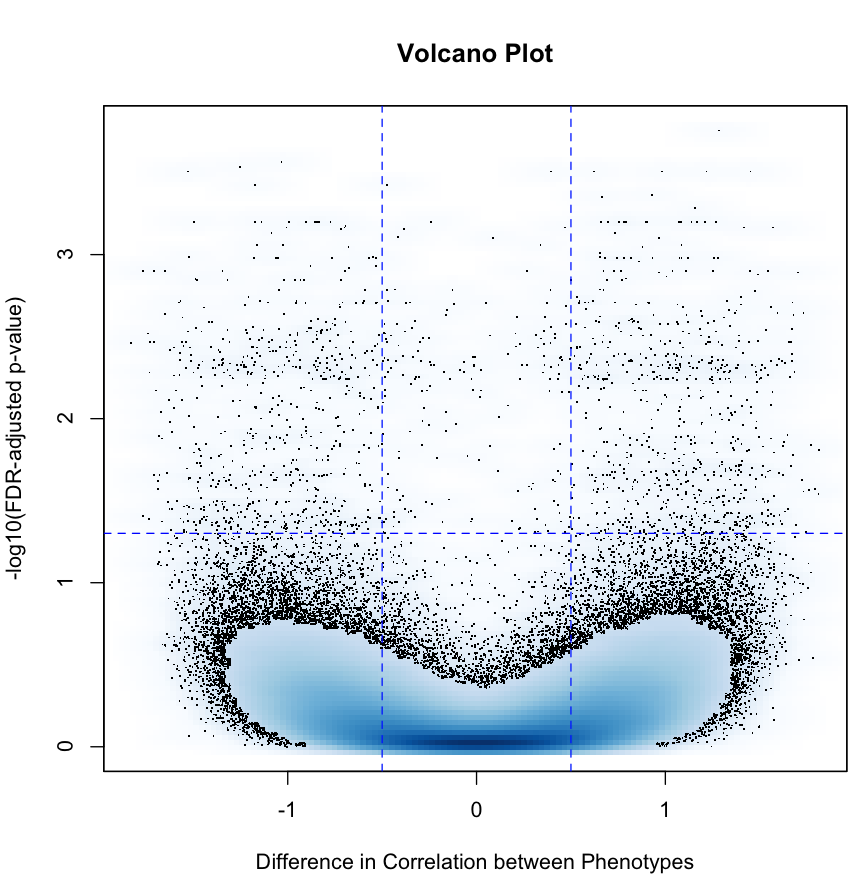
**

Figure 4: Example volcano plot

**Step4: Filter Results**

The ProcessResults() function filters the results by FDR p-values (default set at 0.10) and by the absolute value difference of the gene-metabolite Spearman correlation (default set at 0.50) between the two groups. The output is a list of gene-metabolite pairs and gene-metabolite Spearman correlations for each of the two groups.

The CorrHeatmap() produces a clustered heatmap of these correlations (Figure 5).

**
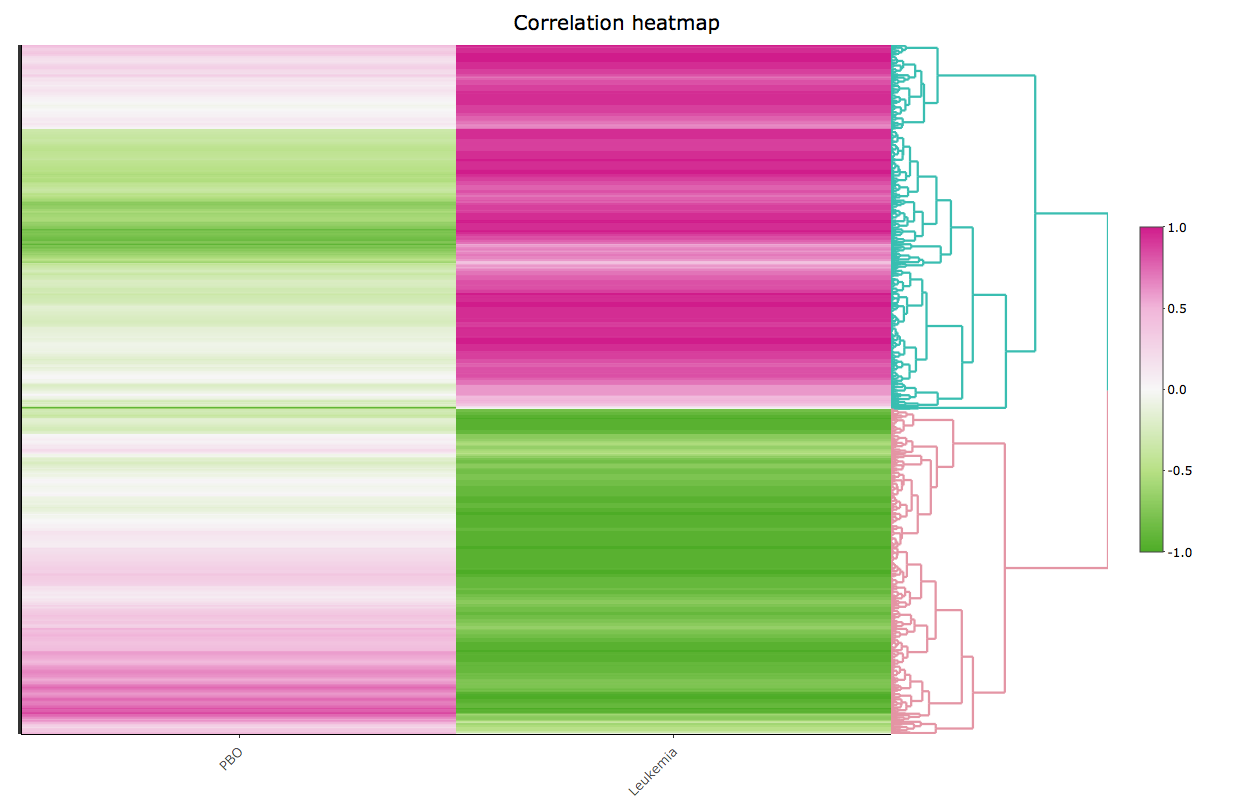
**

Figure 5: Example gene-metabolite correlation heatmap

**Step5: Visualize and Export Results**

A PlotGMPair() function produces a scatterplot of a user-selected gene-metabolite pair, which is color-coded by phenotype (Fig. 6)


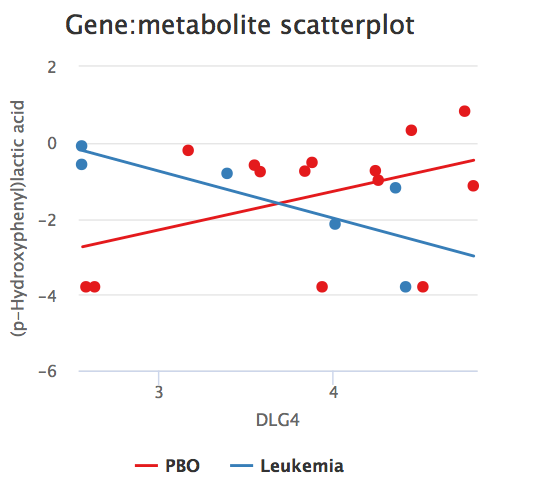


Figure 6: Example of gene-metabolite scatterplot

Importantly, most plots generated in IntLIM use Highcharter (http://jkunst.com/highcharter/) and Plotly [3] (https://plot.ly), which enables interactive visualization and allows users to promptly assess the effect of changing parameters on analysis results and accelerating discovery of phenotype-specific gene-metabolite pairs. This will greatly allow the workflow to be accessible to non-computational biologists.

The OutputData() and OutputResults() function produces tables of data and results of the analyses into zipped CSV files.

**ShinyApp User Interface**

A Shiny App embedded in the package provides a user-friendly interface for running IntLIM (<https://shiny.rstudio.com>) (Fig 7). The App calls functions from the R package so includes all the features describe above. These features include allowing users to input and observe distributions of transcriptomic and metabolomic data (Figure 7A), to filter input data (Figure 7A), to produce distribution of adjusted p-values for interaction coefficients (Figure 7B), to produce volcano plots of Spearman correlations/-log10(FDR-adjusted p-value) (Figure 7B), to produce a heatmap of gene-metabolite correlations for the two selected groups (Figure 7C), and to produce scatter-plots of select gene-metabolite pairs (Figure 7D). The App can be called by typing “runIntLIMApp()” in the R console or RStudio.


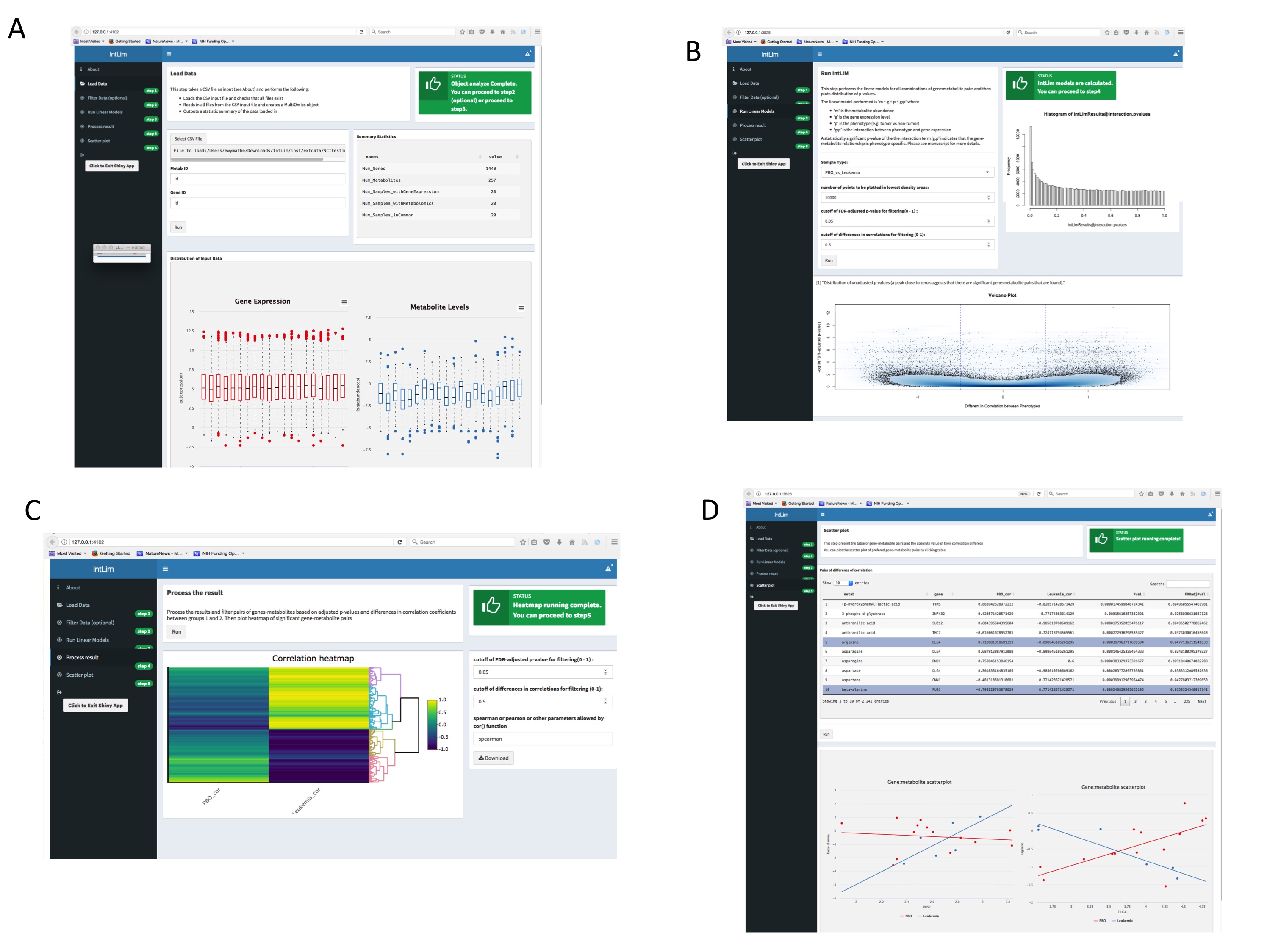


Figure 7: Example of Shiny App. A. Inputting and filtering data. B. Running IntLIM model and observing p-value distribution and volcano plots. C. Observing heatmap of gene-metabolite correlations. D. Scatterplots of select gene-metabolite.

**Vignettes**

The IntLIM Github repository (<https://github.com/mathelab/IntLIM>) includes a vignette with a test data set, which includes a subset of gene and metabolite levels from the original NCI-60 cell line data[4]. This reduced dataset allows the user to work through the steps of the workflow. For the data analyzed in this publication, the NCI-60 cell line data and breast cancer data are also available via the IntLIM GitHub repository.

References

1. Wickham H, Chang W: **devtools: Tools to make developing R code easier**. *R package version* 2015, **1**(0).

2. Hernandez-Ferrer C, Ruiz-Arenas C, Beltran-Gomila A, González JR: **MultiDataSet: an R package for encapsulating multiple data sets with application to omic data integration**. *BMC bioinformatics* 2017, **18**(1):36.

3. Sievert C, Parmer C, Hocking T, Chamberlain S, Ram K, Corvellec M, Despouy P: **plotly: Create Interactive Web Graphics via’plotly. js’. 2016. R package version 3.6. 0**. In*.*

4. Su G, Burant CF, Beecher CW, Athey BD, Meng F: **Integrated metabolome and transcriptome analysis of the NCI60 dataset**. *BMC bioinformatics* 2011, **12**(1):S36.
