# Supplementary material for: IntLIM: integration using linear models of metabolomics and gene expression data
Source: BMC Bioinformatics. 2018 Mar 5;19:81. doi: 10.1186/s12859-018-2085-6 (PMC5838881; doi:10.1186/s12859-018-2085-6)
Supplement: Supplementary file 3 — Figure S1. Preliminary analysis of filtered NCI-60 data involving 14 breast/prostrate/ovarian cancer (BPO) lines and 6 leukemia cell lines with 220 filtered metabolites and 16,188 genes. A) Distribution of normalized (Metabolon method) metabolite abundances among NCI-60 cell lines. B) Distribution of normalized (MAS5 algorithm) gene expression data. C, D) Principal component analysis of metabolomics and gene expression data, respectively. In the IntLIM package Rshiny app, these plots are interactive and hovering over points will provide information on those points (e.g. sample names). (PDF 555 kb) [file 12859_2018_2085_MOESM3_ESM.pdf]

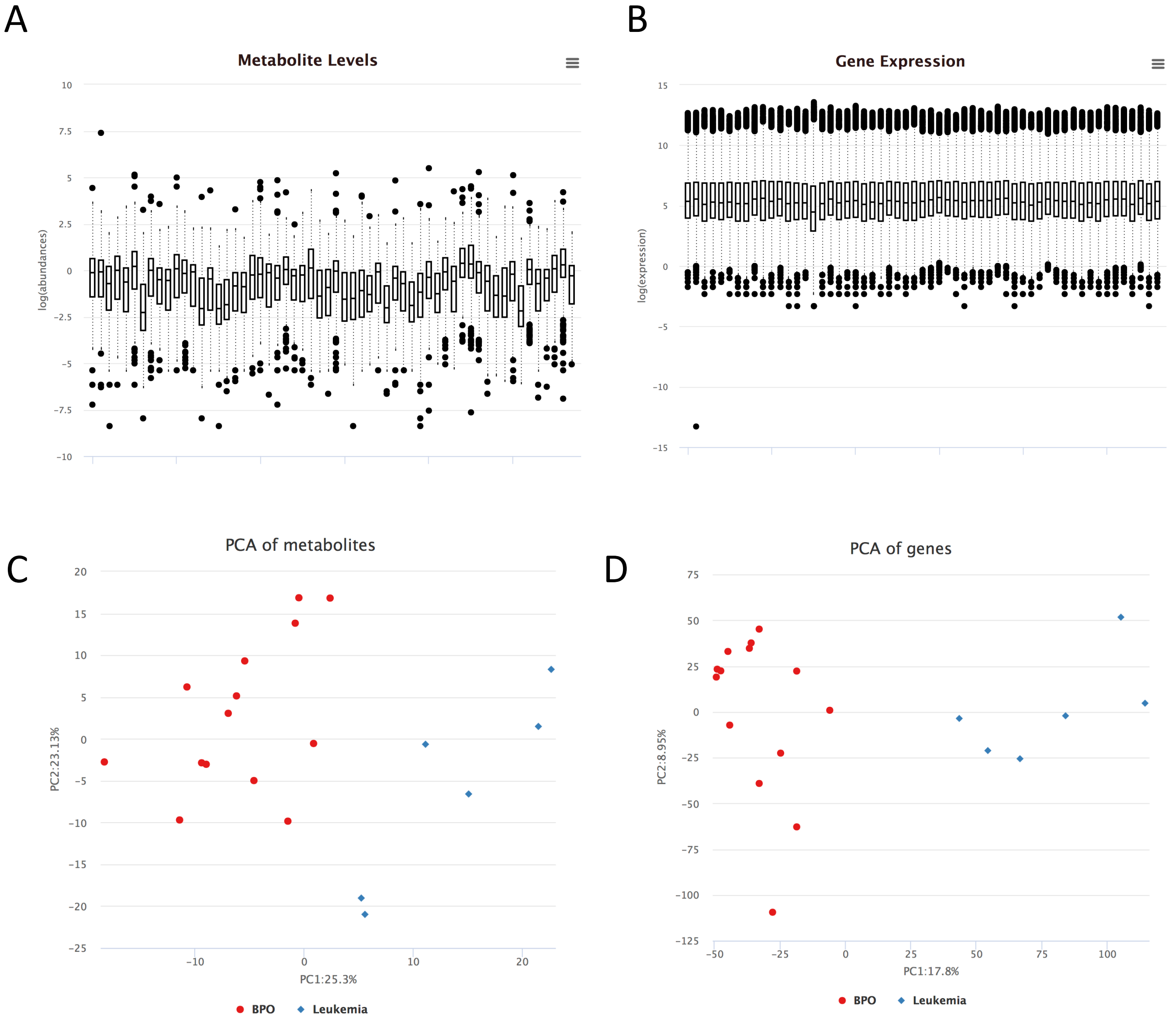

Figure S1: Preliminary analysis of filtered NCI-60 data involving 14 breast/prostrate/ovarian cancer (BPO) lines and 6 leukemia cell lines with 220 filtered metabolites and 16,188 genes . A) Distribution of normalized (Metabolon method) metabolite abundances among NCI-60 cell lines. B) Distribution of normalized (MAS5 algorithm) gene expression data. C, D) Principal component analysis of metabolomics and gene expression data, respectively. In the IntLIM package Rshiny app, these plots are interactive and hovering over points will provide information on those points (e.g. sample names).
