# Supplementary material for: IntLIM: integration using linear models of metabolomics and gene expression data
Source: BMC Bioinformatics. 2018 Mar 5;19:81. doi: 10.1186/s12859-018-2085-6 (PMC5838881; doi:10.1186/s12859-018-2085-6)
Supplement: Supplementary file 5 — Table S1. NCI-60 Data pathway analysis results of genes. Ingenuity Pathway Analysis Canonical Pathways from Genes involved in Gene-Metabolite Pairs of the Leukemia Correlated Cluster and Leukemia Anti-Correlated Cluster. P-values are all calculated from right-tailed Fisher’s Exact Test. (PDF 56 kb) [file 12859_2018_2085_MOESM5_ESM.pdf]

Table S1: NCI-60 Data pathway analysis results of genes. Ingenuity Pathway Analysis Canonical Pathways from Genes involved in Gene-Metabolite Pairs of the Leukemia Correlated Cluster and Leukemia Anti-Correlated Cluster. P-values are all calculated from right-tailed Fisher's Exact Test.

**Leukemia Correlated Cluster**			**Leukemia Anti-Correlated Cluster**		
Pathway	***p-value***	***Overlap***	***Pathway***	***p-value***	***Overlap***
Acute Phase Response Signaling	2.21E-04	6.5% (11/170)	eNOS Signaling	3.06E-04	5.5% (10/181)
1D-myo-inositol Hexakisphosphate Biosynthesis V (from Ins(1,3,4)P3)	9.16E-04	66.7% (2/3)	CREB Signalling in Neurons	5.08E-04	5.2% (10/193)
Hepatic Fibrosis/Hepatic Stellate Cell Activation	1.55E-04	5.5% (10/183)	dTMP De Novo Biosynthesis	9.55E-04	21.4% (3/14)
CDK5 Signaling	1.84E-04	7.1% (7/99)	Huntington's Disease Signaling	1.02E-03	4.4% (11/249)
PAK Signaling	2.06E-04	6.9% (7/101)	P2Y Purigenic Receptor Signaling Pathway	1.12E-03	5.6% (8/143)
