# Supplementary material for: IntLIM: integration using linear models of metabolomics and gene expression data
Source: BMC Bioinformatics. 2018 Mar 5;19:81. doi: 10.1186/s12859-018-2085-6 (PMC5838881; doi:10.1186/s12859-018-2085-6)
Supplement: Supplementary file 6 — Figure S2. Preliminary analysis of filtered breast cancer data involving 108 samples (61 tumor and 47 non-tumor) with 379 metabolites and 18,228 genes. A, B) Distribution of normalized metabolite levels (Metabolon method) and RMA-normalized gene expression levels for all samples, respectively. C,D) Principal component analysis of metabolomics and gene expression data, respectively. In the IntLIM package Rshiny app, these plots are interactive and hovering over points will provide information on those points (e.g. sample names). (PDF 543 kb) [file 12859_2018_2085_MOESM6_ESM.pdf]

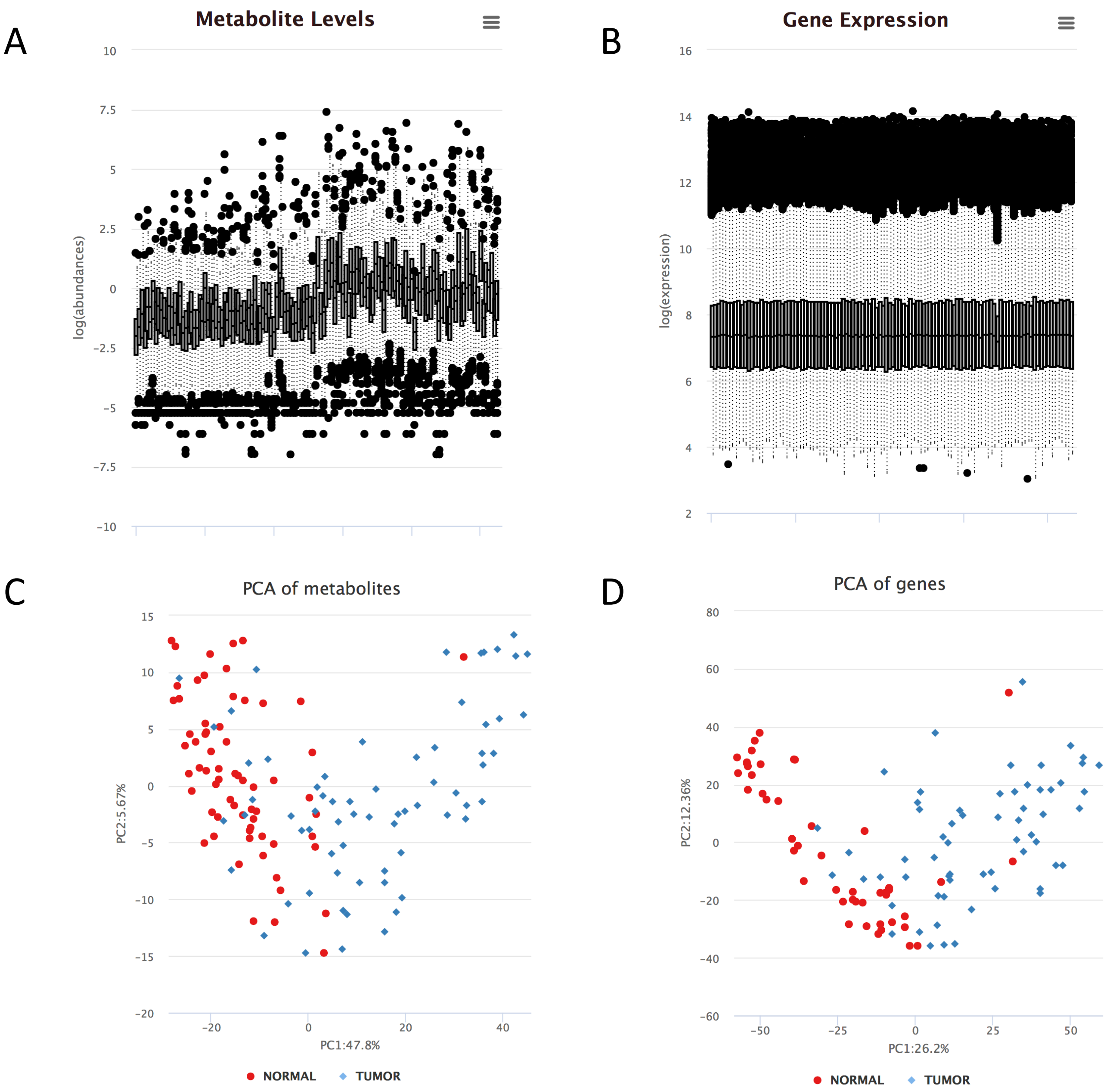

Figure S2: Preliminary analysis of filtered breast cancer data involving 108 samples (61 tumor and 47 non-tumor) with 379 metabolites and 18,228 genes. A, B) Distribution of normalized metabolite levels (Metabolon method) and RMA-normalized gene expression levels for all samples, respectively. C,D) Principal component analysis of metabolomics and gene expression data, respectively. In the IntLIM package Rshiny app, these plots are interactive and hovering over points will provide information on those points (e.g. sample names).
