# Supplementary material for: IntLIM: integration using linear models of metabolomics and gene expression data
Source: BMC Bioinformatics. 2018 Mar 5;19:81. doi: 10.1186/s12859-018-2085-6 (PMC5838881; doi:10.1186/s12859-018-2085-6)
Supplement: Supplementary file 8 — Table S2. Breast Cancer Data pathway analysis results of genes. Ingenuity Pathway Analysis Canonical Pathways from Genes involved in Gene-Metabolite Pairs of the Tumor Correlated Cluster and Tumor Anti-Correlated Cluster. P-values are all calculated from right-tailed Fisher’s Exact Test. (PDF 55 kb) [file 12859_2018_2085_MOESM8_ESM.pdf]

Table S2: Breast Cancer Data pathway analysis results of genes. Ingenuity Pathway Analysis Canonical Pathways from Genes involved in Gene-Metabolite Pairs of the Tumor Correlated Cluster and Tumor Anti-Correlated Cluster. P-values are all calculated from right-tailed Fisher's Exact Test.

**Tumor Correlated Cluster**			**Tumor Anti-Correlated Cluster**		
Name	***p-value***	***Overlap***	***Name***	***p-value***	***Overlap***
Oxidative Phosphorylation	2.00E-15	16.5% (18/109)	Hepatic Fibrosis/Hepatic Stellate Cell Activation	1.21E-06	8.7% (16/183)
Mitochondrial Dysfunction	4.98E-14	11.7% (20/171)	FAK Signaling	3.56E-05	10.1% (10/99)
Protein Ubiquitination Pathway	5.50E-04	4.2% (11/265)	Actin Cytoskeleton Signaling	7.28E-05	6.6% (15/227)
GDP-mannose Biosynthesis	2.27E-03	33.3% (2/6)	Signaling by Rho Family GTPases	1.94E-04	6.0% (15/248)
Pyridoxal 5'-phosphate Salvage Pathway	9.01E-03	6.2% (4/65)	Circadian Rhythm Signaling	5.18E-04	15.2% (5/33)
