# Supplementary material for: IntLIM: integration using linear models of metabolomics and gene expression data
Source: BMC Bioinformatics. 2018 Mar 5;19:81. doi: 10.1186/s12859-018-2085-6 (PMC5838881; doi:10.1186/s12859-018-2085-6)
Supplement: Supplementary file 9 — Table S3. Breast Cancer Data pathway analysis results of metabolites. Ingenuity Pathway Analysis Canonical Pathways from Metabolites involved in Gene-Metabolite Pairs of the Tumor Correlated Cluster. P-values are all calculated from right-tailed Fisher’s Exact Test. (PDF 53 kb) [file 12859_2018_2085_MOESM9_ESM.pdf]

Table S3: Breast Cancer Data pathway analysis results of metabolites. Ingenuity Pathway Analysis Canonical Pathways from Metabolites involved in Gene-Metabolite Pairs of the Tumor Correlated Cluster. P-values are all calculated from right-tailed Fisher's Exact Test.

**Tumor Correlated Cluster**			**Tumor Anti-Correlated Cluster**		
Name	***p-value***	***Overlap***	***Name***	***p-value***	***Overlap***
tRNA charging	1.33E-11	39.5% (17/43)	tRNA charging	1.09E-11	41.9% (18/43)
Purine Ribonucleosides Degradation to Ribose-1-phosphate	8.77E-07	58.3% (7/12)	Superpathway of Citrulline Metabolism	6.83E-05	33.3% (8/24)
Purine Nucleotides Degradation II (Aerobic)	1.71E-05	41.2% (7/17)	Urea Cycle	1.20E-04	42.9% (6/14)
Guanosine Nucleotides Degradation II	1.01E-04	50.0% (5/10)	Purine Nucleotides Degradation II (Aerobic)	4.22E-04	35.3% (6/17)
Adenosine Nucleotides Degradation III	1.78E-04	45.5% (5/11)	Purine Ribonucleosides Degradation to Ribose-1-phosphate	5.50E-04	41.7% (5/12)
